# Supplementary material for: Olive Pomace Oil versus High Oleic Sunflower Oil and Sunflower Oil: A Comparative Study in Healthy and Cardiovascular Risk Humans
Source: Foods. 2022 Jul 22;11(15):2186. doi: 10.3390/foods11152186 (PMC9331821; doi:10.3390/foods11152186)
Supplement: Supplementary file 1 [file foods-11-02186-s001.zip › foods-1804320-supplementary.pdf]

Table S1. Chemical composition of olive pomace oil (OPO), high oleic sunflower oil (HOSO), sunflower oil (SO) and corn oil (CO).

	OPO	HOSO	SO	CO
**Fatty acids (%)**				
C 12:0 (Lauric acid)	-	-	-	0.01
C 14:0 (Myristic acid)	0.02	0.0	0.1	0.04
C 16:0 (Palmitic acid)	11.63	4.2	6.4	10.60
C 16:1 (Palmitoleic acid)	0.98	0.1	0.2	0.15
C 17:0 (Margaric acid)	0.07	0.0	0.1	0.07
C 17:1 (Margaroleic acid)	0.11	0.1	0.1	0.05
C 18:0 (Stearic acid)	2.83	3.3	3.6	2.03
C 18:1 (Oleic acid)	71.01	76.5	29.6	35.36
C 18:2 (Linoleic acid)	11.5	13.9	58.6	50.52
C 20:0 (Araquic acid)	0.49	0.3	0.3	0.48
C 18:3 (Linolenic acid)	0.76	0.1	0.1	0.04
C 20:1 (Eicosenoic acid)	0.34	0.2	0.2	0.31
C 22:0 (Behenic acid)	0.19	1.0	0.7	0.17
C 22:1 (Erucic acid)	-	-	< 0.01	< 0.01
C 24:0 (Lignoceric acid)	0.07	0.3	0.2	0.17
Trans Oleic (*t*-C18:1)	0.32	0.0	0.0	0.02
Trans Linoleic + *Trans* Linolenic (*t*-C18:2 + *t*-C18:3)	0.1	0.2	0.4	1.15
**Squalene (ppm)**				
Squalene (ppm)	799	87	117	548
**Tocopherols (mg/kg)**				
α-Tocopherol (Vit. E)	357	420	518	33
β-Tocopherol	<2	40	44	<2
◎-Tocopherol	32	72	<2	205
δ -Tocopherol	<2	51	<2	6
**Tocotrienols (mg/kg)**				
A-Tocotrienol	-		-	10

β-Tocotrienol	-		-	<2
ϒ-Tocotrienol	-		-	5
Δ-Tocotrienol	-		-	<2
**Sterols (%)**				
Cholesterol	0.1	0.1	0.1	0.21
Brassicasterol	<0.1	<0.1	<0.1	0.62
24-Methylcholesterol	0.2	0.2	0.3	0.92
Campesterol	3.2	8.7	8.7	20.75
Campestanol	0.1	0.1	0.1	0.92
Stigmasterol	1.2	8.4	7.8	6.85
Δ7-Campesterol	<0.1	2.9	2.7	<0.10
Δ5,23-Stigmastadienol	0.8	0.2	0.1	<0.10
Clerosterol	1.5	0.8	0.7	0.70
β -Sitosterol	86.1	54.1	54.6	61.09
Sitostanol	1.7	0.6	0.5	2.11
Δ5-Avenasterol	1.6	3.6	3.4	3.90
Δ5,24-Stigmastadienol	2	1.1	1	0.51
Δ7-Stigmastenol	0.4	13.5	14.5	0.63
Δ7-Avenasterol	1	5.7	5.5	0.78
Δ -Sitosterol apparent	93.8	60.5	60.3	68.31
Total Sterols (ppm)	2839	3040	3315	8962.0
**Triterpenic alcohols (mg/kg)**				
Erythrodiol + Uvaol	886.6	<1.0	<1.0	<1.0
**Phenols (mg/kg)**				
Total phenols	< 1.0	<1.0	< 1.0	< 1.0
**Triterpenic acids (mg/kg)**				
Oleanolic acid	187.3	<1.0	<2.0	<2.0
Ursolic acid	6.9	<1.0	<2.0	<2.0
Maslinic acid	2.3	<1.0	<2.0	<2.0
**Aliphatic alcohols (mg/kg)**				
C22+C24+C26+C28	978	32	26	29

Table S2. Effects of olive pomace oil (OPO), high oleic sunflower oil (HOSO) and sunflower oil (SO) on biochemical parameters.

	**Normocholesterolemic**			**Hypercholesterolemic**			***p* value**		
(%)	**OPO** *n=65*	**HOSO** *n=34*	**SO** *n=31*	**OPO** *n=67*	**HOSO** *n=30*	**SO** *n=37*	**Oil**	**N/H**	**Oil* N/H**
**Total cholesterol**	-1.4 ± 1.2	4.9 ± 2.0	1.1 ± 1.8	-0.3 ± 1.0	2.2 ± 2.0	-0.1 ± 1.6	0.017	0.479	0.420
**Triglycerides**	-1.5 ± 2.9	10.4 ± 6.1	-1.8 ± 5.0	13.8 ± 9.1	4.5 ± 7.0	6.9 ± 5.7	0.707	0.955	0.314
**HDL-cholesterol**	0.4 ± 1.3	2.3 ± 1.8	0.8 ± 2.2	2.4 ± 1.5	3.3 ± 2.1	-2.0 ± 1.9	0.094	0.958	0.468
**LDL-cholesterol**	-1.6 ± 1.8	4.2 ± 2.2	3.4 ± 2.8	-1.3 ± 1.4	4.1 ± 3.5	1.0 ± 2.2	0.018	0.643	0.892
**VLDL-cholesterol**	-2.6 ± 3.1	11.1 ± 6.2	-2.9 ± 4.7	13.8 ± 9.4	3.7 ± 7.0	5.7 ± 5.8	0.658	0.920	0.237
**Apo A1**	0.5 ± 1.2	1.9 ± 1.6	2.9 ± 1.7	3.5 ± 1.0	1.2 ± 1.7	1.0 ± 1.3	0.969	0.901	0.132
**Apo B**	2.7 ± 1.6	0.7 ± 2.2	9.5 ± 2.3	1.7 ± 1.6	-0.5 ± 2.5	9.0 ± 1.9	<0.001	0.640	0.965
**Apo B / Apo A1**	1.4 ± 2.0	-2.5 ± 2.6	6.3 ± 2.7	-1.2 ± 2.2	-2.0 ± 2.6	11.3 ± 2.6	<0.001	0.726	0.303
**LDL/HDL**	-1.1 ± 2.1	3.0 ± 2.8	4.3 ± 3.7	-2.2 ± 1.9	1.7 ± 3.7	4.3 ± 3.0	0.056	0.731	0.971
**CT/HDL**	-1.3 ± 1.2	1.8 ± 1.7	1.3 ± 2.2	-1.6 ± 1.3	-0.5 ± 2.1	2.9 ± 2.0	0.089	0.694	0.595

Values represent mean ± standard error of mean. The rate of change was calculated from [(post-treatment value – pre-treatment value)/ pre-treatment value] and expressed as percentage. A linear mixed model was used for data analysis. *p*-value in the first column represents the treatment effect (OPO, HOSO or SO), in the second column the group effect [normocholesterolemic (N) and hypercholesterolemic (H)], and in the last column the interaction of treatment and group. Significance level was set at $p<0.05$. Apo: apolipoprotein.

Table S3. Effects of olive pomace oil (OPO), high oleic sunflower oil (HOSO) and sunflower oil (SO) on blood pressure.

	Normocholesterolemic			Hypercholesterolemic			*p* value		
(%)	**OPO** *n=65*	**HOSO** *n=34*	**SO** *n=31*	**OPO** *n=67*	**HOSO** *n=30*	**SO** *n=37*	**Oil**	**N/H**	**Oil* N/H**
**Systolic BP**	-0.1 ± 0.6	-2.0 ± 1.2	-0.5 ± 0.9	-0.4 ± 0.7	-0.4 ± 1.4	-0.1 ± 1.2	0.532	0.478	0.587
**Diastolic BP**	-0.4 ± 1.0	-1.0 ± 1.6	0.0 ± 1.2	0.1 ± 0.8	3.3 ± 1.9	-0.5 ± 1.1	0.652	0.148	0.212

Values represent mean ± standard error of mean. The rate of change was calculated from [(post-treatment value – pre-treatment value)/ pre-treatment value] and expressed as percentage. A linear mixed model was used for data analysis. *p*-value in the first column represents the treatment effect (OPO, HOSO or SO), in the second column the group effect [normocholesterolemic (N) and hypercholesterolemic (H)], and in the last column the interaction of treatment and group. Significance level was set at $p<0.05$.
